# Impacts of psychological wellbeing with HIV/AIDS and cancer among sexual and gender minorities: A systematic review and meta-analysis

**DOI:** 10.3389/fpubh.2022.912980

**Published:** 2022-11-18

**Authors:** Alex Siu Wing Chan, Lok Man Leung, Jane Siu Fan Li, Jacqueline Mei Chi Ho, Hon Lon Tam, Wing Leung Hsu, April Nicole On Sang Iu, Patrick Ming Kuen Tang, Elsie Yan

**Affiliations:** ^1^Department of Applied Social Sciences, Faculty of Health and Social Sciences, The Hong Kong Polytechnic University, Kowloon, Hong Kong SAR, China; ^2^Department of Anatomical and Cellular Pathology, State Key Laboratory of Translational Oncology, The Chinese University of Hong Kong, Hong Kong, Hong Kong SAR, China; ^3^Faculty of Health and Social Sciences, School of Nursing, Hong Kong Polytechnic University, Kowloon, Hong Kong SAR, China; ^4^Faculty of Medicine, Nethersole School of Nursing, The Chinese University of Hong Kong, Kowloon, Hong Kong SAR, China; ^5^Aceso Medical Centre, Hong Kong, Hong Kong SAR, China; ^6^Department of Pharmacy, Health and Well-being, University of Sunderland, Sunderland, United Kingdom; ^7^Department of Psychology, Faculty of Medicine, Health and Human Sciences, Macquarie University, Sydney, NSW, Australia

**Keywords:** HIV/AIDS, cancer, psychological wellbeing, sexual minorities, patient care, LGBT, health care strategies

## Abstract

**Background:**

The agony and economic strain of cancer and HIV/AIDS therapies severely impact patients' psychological wellbeing. Meanwhile, sexual minorities experience discrimination and mental illness. LGBT individuals with cancer and HIV/AIDS play two roles. It is important to understand and examine this groups mental wellbeing.

**Objective:**

The purpose of this study is to synthesize current studies on the impact of HIV/AIDS and cancer on LGBT patients' psychological wellbeing.

**Methods:**

This research uses a systematic literature review at first and later stage a meta-analysis was run on the same review. In this study, data from Google academic and Web of Science has been used to filter literature. PRISMA 2020 Flow Diagram seeks research on LGBT cancer and HIV/AIDS patients. The above sites yielded 370 related papers, some of which were removed due to age or inaccuracy. Finally, meta-analyses was done on 27 HIV/AIDS and 33 cancer patients's analyse.

**Results:**

The research included 9,898 LGBT cancer sufferers with AIDS and 14,465 cancer sufferers with HIV/AIDS. Using meta-analysis, we discovered the gap in psychological wellbeing scores between HIV/AIDS LGBT and non-LGBT groups ranged from −10.86 to 15.63. The overall score disparity between the HIV/AIDS LGBT and non-LGBT groups was 1.270 (95% CI = 0.990–1.560, *Z* = 86.58, *P* < 0.1). The disparity in psychological wellbeing scores between cancer LGBT group and general group varies from −8.77 to 20.94 in the 34 papers examined in this study. Overall, the psychological wellbeing score disparity between the cancer LGBT subset and the general group was 12.48 (95% CI was 10.05–14.92, Test *Z*-value was 268.40, *P*-value was <0.1).

**Conclusion:**

Inflammation and fibrosis in HIV/AIDS and cancer sufferers adversely affect their psychological wellbeing.

## Introduction

Non-Hodgkin lymphoma, Kaposi's sarcoma, and cervical cancer, which are known as AIDS-defining cancers (ADCs), occur more frequently in HIV/AIDS patients than in HIV-negative individuals (1, 2). In other words, the HIV/AIDS conditions contribute to the development of these cancers in HIV-positive individuals. Aside from this, there is evidence that HIV/AIDS patients are at a higher risk for developing certain non-AIDS-defining cancers (NADCs), despite the fact that there is no known direct pathological relationship between HIV/AIDS and these cancers, unlike the relationship between HIV/AIDS and ADCs (1).

In addition to being one of these NADCs, prostate cancer is the second leading cause of cancer death among men in the United States. While the effects of prostate cancer detection and treatment on the mental health of sexual minorities, such as males who are sexually attracted to males or transgender females, remain unknown, there is a growing body of evidence that suggests that these treatments may be beneficial (3, 4). The infrequency with which information on patients' sexual orientation is collected makes it difficult to conduct research on this population. In fact, several epidemiological studies involving prostate cancer patients from sexual minorities have demonstrated varying rates of prostate cancer screening, diagnosis, and treatment (5).

According to a number of qualitative studies, sexual minority communities have substantial cancer health inequalities (6, 7). As a consequence of differences in sexual behavior, social support networks, and links to the health sector, sexual minorities' experiences with prostate cancer are distinct and need individualized medical attention (8, 9). Notably, sexual minorities among prostate cancer patients were found to have more severe health-related quality of life consequences than heterosexual male patients: having weaker support networks, experiencing greater mental disturbance due to sexual problems such as undefined fields after therapies, being excluded from the health sector, and expressing greater dissatisfaction with therapies (10).

Additionally, few oncology professionals have received training on how to best serve the needs of Sexual and Gender Minority (SGM) patients, and few cancer centers have implemented policies or regular procedures to gather sexual orientation and gender identity information in the electronic medical record, utilize gender-neutral language on forms, provide SGM-specific support services, and/or mandate SGM cultural humility training for all personnel (11). Until doctors receive adequate training on the clinical and behavioral requirements of SGM patients, patients will continue to be responsible for teaching their physicians how to care for them, leading to inadequate treatment and perhaps reinforcing the stigmatizing actions of clinicians (12, 13).

Consequently, the effects of HIV/AIDS and cancer on the mental health of HIV/AIDS-related cancer patients are deserving of study. Although there has historically been a paucity of literature on sexual minorities among cancer patients, there has been a substantial increase in research on the topic in recent years, solidifying its position as an important area of inquiry. This study aims to synthesize current research on the impact of HIV/AIDS and cancer on the psychological wellbeing of LGBT patients.

## Literature review

### Sexual minorities

When it comes to defining sexual minorities, because they are a notion brought and transferred from outside, the academic field largely agrees with the United Nations Development Programme's 2016 survey report on the survival of sexual minorities in refer to those belonging to minorities in terms of sexual orientation, gender identity and gender expression (14). Sexual orientation refers to individuals of a particular gender who are the subject of emotional inclination and sex drive. For instance, if the target of emotional inclination and sex drive is homosexual, it is referred to as homosexual; if the target is both gay and heterosexual, it is referred to as bisexual (15–17).

Gender identity refers to an individual's emotional proclivity and psychological identification with a certain gender. Transgender individuals, for instance, identify as females when their biological gender at birth is male, despite the fact that their biological gender was female, or as males when their biological gender was female, thus constituting a minority in terms of gender identity (18, 19). Gender expression is the process of expressing one's gender through clothing, grooming, and conduct. For instance, males who dress up as females or females who dress up as males are considered minority groups in terms of gender expression and are referred to as transvestites (20). Nevertheless, the academic world turns a blind eye to these communities (21, 22). Sexual minorities, as defined above, primarily comprise homosexuals, bisexuals, transgenders, and intersexual. As a result, some academics think that sexual minorities are sexual orientation minorities in comparison to heterosexual people, including lesbians, gay men, bisexuals, and transgender people (LGBT) (23). Its flaw is that its restricted reach excludes various sexual minorities and does not promote the rights and welfare of diverse groups based on sexual orientation, gender identity, and gender expression as civilization develops (24–26).

### Impacts on psychological wellbeing among cancer patients

Individuals will experience significant pain in their body, mind, and interpersonal connection following cancer diagnosis and therapy, leading to a variety of mental conditions (27, 28). Such biological and cognitive shifts have a detrimental effect on the psychological wellbeing and prognosis of breast cancer sufferers, perpetuating the cycle. Numerous empirical researches conducted domestically and overseas demonstrates that good psychological tools benefit the psychological wellbeing of Anzheng sufferers (29, 30). Deimling et al. (31) and Sardella et al. (32) discovered that the degree of psychological optimism in elderly cancer patients may predict disease progression. According to Sitanggang et al. (33), cancer sufferers with a high level of expectation to be more pleased and acknowledged in their marriage (partnership). Putri and Makiyah (34) discovered that cancer sufferers with a poor ego are more receptive to more invasive treatment approaches.

Simultaneously, the greater the level of self-esteem, the more satisfied patients are with their therapy (35). Self-esteem is a critical protective element for cancer sufferers' psychological wellbeing (36). Ristevska-Dimitrovska and Batic (37) discovered that sufferers with improved psychological wellbeing also improved their quality of life, while their functioning improved. While successfully relieved cancer-related symptoms, Ristevska-Dimitrovska and Batic (37) also noted that adaptability was a potential mechanism against depression and psychological illnesses (38). Carver stresses that early sufferers who have a high level of hopeful may not only have more realistic predictions about their state, but also have a more favorable impact on postoperative recovery (39, 40). Garner and de Visser (41) discovered that optimism not only has a significant impact on depression alleviation, but also has an additional impact through social connectedness. To summarize, there is a relationship between cancer and sufferers' psychological wellbeing. While the suffering associated with cancer therapy has a direct impact on patients' psychological wellbeing, the level of patients' psychological wellbeing also has a significant impact on the curative implication and therapy intensity of cancer (42–45).

### Study on psychological wellbeing HIV/AIDS patients

Currently, there is no real treatment for HIV/AIDS (46). When infected, it will be with you for the rest of your life. It is incapable of eradicating severe infections, which is why AIDS is transmitting throughout the globe. HIV/AIDS cases are rising (47). According to UNAIDS data, 38 million individuals globally are HIV-positive. HIV infected 1 million 700,000 of the globe's newly infected individuals in 2019. Six hundred ninety thousand individuals died of AIDS-related diseases in 2019 (48). According to the existing state of knowledge, the majority of analyses presume that current HIV/AIDS patients suffer from serious mental issues marked by depression and anxiety (49–52). Social discrimination and isolation are significant contributors to psychological wellbeing issues among HIV-positive individuals (53–56).

Discrimination against HIV/AIDS-related groups is primarily motivated by two factors. To begin, a large percentage of AIDS patients are men who have sex with men, intravenous drug users, and commercial sex traders (57). These individuals are frequently not approved by the majority of the general public, and they are viewed as having moral flaws and character flaws. Two, the HIV/AIDS group is highly contagious and poses a threat to others. HIV/AIDS communities are frequently portrayed negatively in news coverage, and are frequently categorized as “dangerous” and “revenge society” (58, 59). With such a social paradigm, it is not only challenging to be completely compassionate and selfless, but also frequently confronts the conundrum of causing more difficulties for oneself by exposing identity (60). As a result of their shame and self-protection, many HIV-positive individuals are hesitant to disclose their infection to their neighbors or even family members (61). Additionally, they reject some individuals access to clinical, mental, and community services. Individuals with HIV have mental issues as a result of their dual physiological and psychological problems, and they may be dealing with mental anxiety, depression, or even the Dutch act (17), which happens regularly (62, 63).

## Methods

This research used a systematic review and meta-analysis as its methodology. The primary techniques of study include literature review, questionnaires, and meta-analysis. Meta-analysis is a mathematical process that integrates the findings of many studies conducted in the same field under similar circumstances. The researcher mostly utilizes stata.16 as the statistical analysis program to generate the meta-analysis table and forest map, and the statistics are obtained from the internet and Google academic. The PRISMA 2020 Flow Diagram is employed to conduct a literature screening for publications involving older men who have sex with men with cancer. The authors gathered a total of 370 relevant papers through Google academic and websites, and eliminated several due to their younger age and imprecise statistical representation. Lastly, the meta-analysis comprised 27 papers on HIV/AIDS and 33 studies on cancer.

### Search strategy

**Figure 1:**
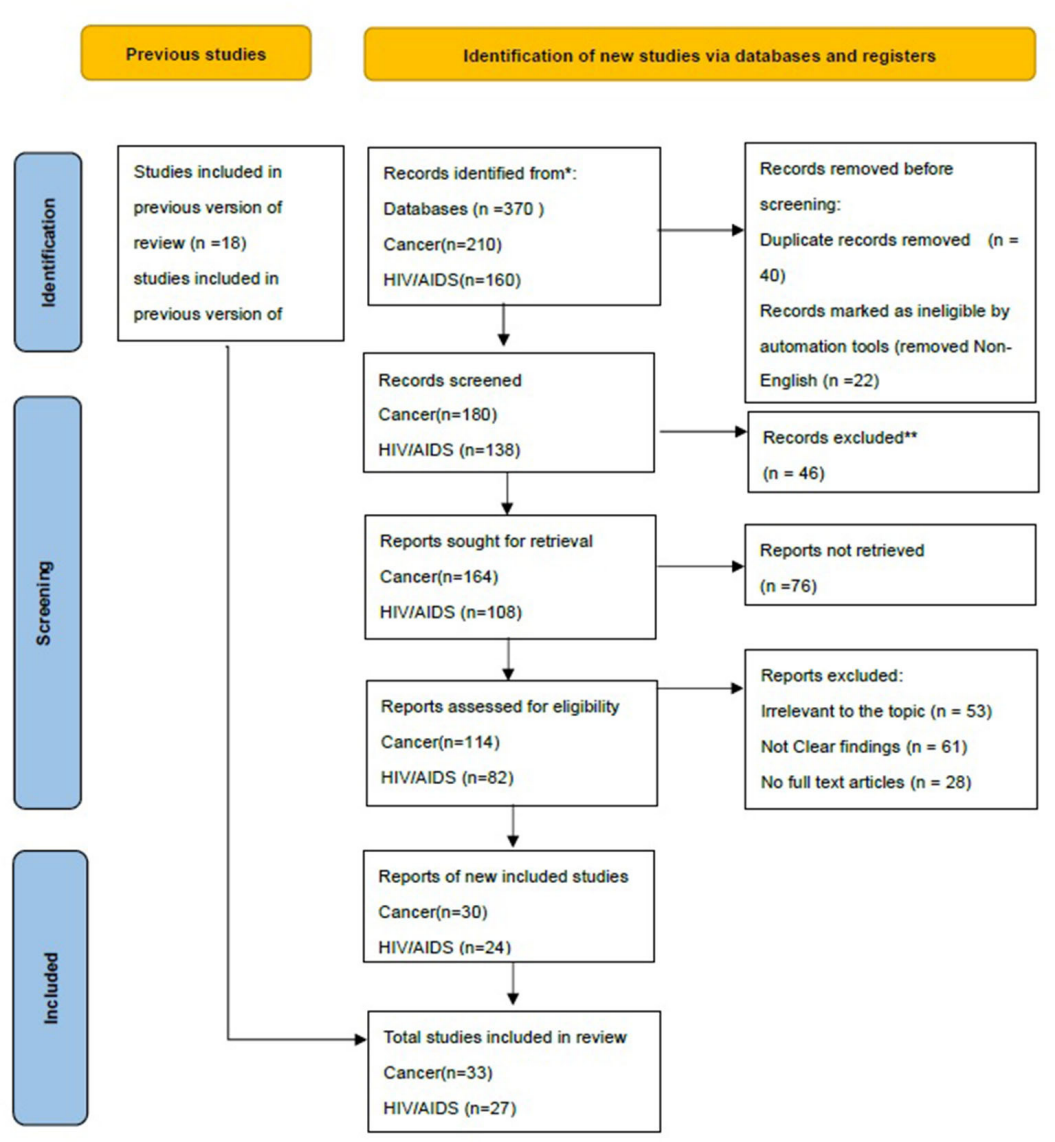
Study selection based on PRISMA 2020 flow diagram.

Numerous researches have been conducted on the psychological wellbeing of HIV/AIDS and Cancer sufferers, but few on the psychological wellbeing of HIV/AIDS and Cancer LGBT sufferers. This research performed a systematic review and meta-analysis of articles published between August 1, 2018 and August 1, 2021 in Google scholarly and Web of Science. Such personal records investigated homosexuality, cancer sufferers, geriatric populations, and psychotherapy treatments. The authors mostly utilizes PRISMA 2020 Flow Diagram (Registered Code: CRD42022314571) to conduct literature searches (Figure 1).

### Study inclusion and exclusion criteria

To conduct research on the effect of cancer and HIV/AIDS on the mental wellbeing of the LGBT community, we must collect data on the psychological wellbeing of gays and cancer sufferers. We solely considered retrospective or prospective observational research and omitted other kinds of literature (like reviewing, editing, case study, consensus therapy, or recommendations). Additionally, research lacking the whole text was eliminated.

All databases were queried using the keywords, and the results were exported to the citation management system EndNote Reference, where the duplicates were removed. The second stage is to review the abstracts and titles of the remaining publications to determine which are relevant to the study at hand. Before evaluating the papers based on the qualifying criteria, three reviewers (ASWC, WLH, and JMCH) conducted a preliminary screening. After title screening, full-text research must meet inclusion and exclusion criteria. The qualifying conditions are shown in Table 1 below.

**Table 1:** Inclusion and exclusion criteria.

	**Inclusion**	**Exclusion**
Participants	• Studies that included adult or older male participants	• Studies that did not clearly state the classification of participants according to their gender
Study type and details	• Studies with transparent findings • Studies with full-text manuscripts • English published studies • Primary and observational studies	• Studies that lacked interpretable and clear findings • Studies without full-text manuscripts • Non-English published articles • Case reports, other systematic reviews, and meta-analyses
Outcome	• Studies evaluating HIV/AIDS and Cancer and impacts of Psychological Wellbeing among Sexual Minorities	• Studies evaluating other outcomes apart from Studies evaluating HIV/ AIDS and Cancer and impacts of Psychological Wellbeing among Sexual Minorities

### Literature selection

Upon removing redundant publications from the bibliographic database's research outcomes, three independent researchers (ASWC, WLH, and JMCH) evaluated the remaining titles and abstracts for papers that may qualify for full-text evaluation. Additionally, the bibliography of publications included in this manner is carefully researched. Following an examination of the raw data and consultation with another researcher (ANOSI), any discrepancies were addressed through conversation.

### Meta-analysis

#### Data collection and extraction

The same scholars finished and inspected data extraction. Gender, cancer, demographic factors (age, gender, sexuality, household income, and geographic area), clinical signs (primarily inflammation and fibrosis), and patient objective records (like psychological wellbeing) are all retrieved (64–67).

#### A meta-analysis of the psychological effects of HIV/AIDS LGBT patients

As per clinical capability at the time of writing, AIDS is an untreatable illness. In the present research, there are limited papers on the impact of inflammation and fibrosis on the psychological wellbeing of AIDS sufferers. Prisma 2020 was used to conduct a search of the literature, some of which had issues including a huge sample size or a lack of clarity in the reporting of findings. Ultimately, twenty-seven papers were chosen for meta-analysis. The following are the findings:

Table 2 summarizes a meta-analysis of the impact of inflammation and fibrosis on the psychological wellbeing of HIV/AIDS sufferers. The SMD column in the table indicates the meta-analysis's associated response value. A comparison experiment was used to perform the meta-analysis. The control group consisted of healthy individuals. The control group consisted of an HIV/AIDS LGBT patient who acted as an inhibitor of HIV/AIDS-related inflammation and fibrosis. Through looking at the average scores for the two factors on psychological wellbeing factors, we may determine if HIV/AIDS-related inflammation and fibrosis have a substantial impact on the psychological wellbeing of LGBT patients. The greater the value, the more dysfunctional the psychological state. SMD one denotes the disparity in scores between the two categories.

**Table 2:** Mental health meta-analysis of HIV/AIDS LGBT patients.

**Author(s) (year)**	**SMD**	**[95% Conf. interval]**	**[95% Conf. interval]**	**% Weight**	**Study quality**
Tomar et al. (2021) (61)	6.41	4.13	8.69	1.6	Good
Philpot et al. (2021) (68)	−6.96	−8.59	−5.33	3.12	Good
Liboro et al. (2021) (69)	10.48	8.94	12.02	3.52	Good
Gonzales et al. (2017) (70)	18.90	17.23	20.56	2.99	Good
Freese et al. (2017) (71)	9.94	8.68	11.21	5.22	Good
Batchelder et al. (2017) (72)	7.24	5.47	9.01	2.66	Moderate
Wilson et al. (2016) (73)	3.94	2.44	5.44	3.72	Good
Rodriguez et al. (2016) (74)	13.67	12.42	14.92	5.34	Good
Liboro et al. (2016) (75)	15.61	17.44	13.77	2.48	Good
Dowshen et al. (2016) (76)	−2.17	−3.75	−0.59	3.34	Good
Swartz et al. (2015) (77)	2.81	1.83	3.79	8.7	Moderate
Lewis et al. (2015) (78)	−3.08	−4.38	−1.79	4.95	Moderate
Jadwin et al. (2015) (79)	6.39	4.86	7.93	3.52	Good
Garland et al. (2014) (80)	2.21	0.84	3.59	4.41	Good
DiNapoli et al. (2014) (81)	−10.86	−12.30	−9.42	4.01	Good
Coulter et al. (2014) (82)	1.10	−1.55	1.55	3.46	Good
Cahill et al. (2014) (83)	−6.42	−8.09	−4.76	3.01	Good
Hergenrather et al. (2013) (84)	−3.94	−5.42	−2.46	3.78	Good
Grey et al. (2013) (85)	−2.11	−4.02	−0.19	2.27	Moderate
Brennan et al. (2013) (86)	−0.40	−2.06	1.27	3	Good
Wight et al. (2012) (87)	−6.88	−8.59	−5.18	2.86	Good
Haile et al. (2011) (88)	1.67	0.28	3.06	4.32	Good
Pantalone et al. (2010) (89)	6.28	4.53	8.04	2.7	Good
Tritt (2010) et al. (90)	3.82	2.26	5.38	3.43	Good
McDowell et al. (2007) (91)	−5.22	−6.51	−3.94	5.03	Good
Countenay et al. (2006) (92)	1.69	0.01	3.37	2.95	Good
Wilson et al. (2004) (93)	−5.92	−7.43	−4.40	3.63	Good
Overall, IV	1.270	0.990	1.560	100	

Test of overall effect = 0: z = 86.58, p < 0.0001.

The majority of the research reports a favorable score disparity. This demonstrates that AIDS-related inflammation and fibrosis will have a detrimental effect on the psychological wellbeing of LGBT AIDS sufferers. After analyzing 27 publications on the psychological wellbeing of the AIDS LGBT community, we discovered that the gap in psychological wellbeing scores between the HIV/ AIDS LGBT community and the general population ranges from −10.86 to 15.63. The overall findings indicated a 1.270 point gap in psychological wellbeing scores between the AIDS LGBT population and the general population (95% confidence interval 0.990–1.560, *Z* = 86.58 and *P* < 0.001).

Although the mental health measurement methods or scales used in these studies are different in different kinds of literature, as a meta-analysis, this study did not consider different intervention therapies. As can be seen from the above table, the final overall *Z*-value is 86.58 and the *P* < 0.1, which indicates that the average score of mental health in the control group is 1.270 points higher than that in the experimental group. That is, the inflammation and fibrosis of HIV/AIDS have a significant effect on the negative mental health of the LGBT group.

Table 3 summarizes the heterogeneity test findings from 27 research studies. Like the table indicates, the *p*-value is 0.04, which is smaller than 0.001, suggesting heterogeneity. The heterogeneity score is 99.0%, suggesting that available research has a high degree of heterogeneity. Lastly, we can observe that the associated Cochran's *Q*-value is 2,476.47 and the accompanying *p* < 0.1, indicating that inflammation and fibrosis in HIV/AIDS have a substantial detrimental effect on LGBT sufferers' psychological wellbeing.

**Table 3:** Heterogeneity analysis of related studies.

**Measure**	**Value**	***df***	***p*** **-value**
Cochran's Q	2,476.47	26	0.0001
H	9.760	1.000	
*I^2^* (%)	99.0%	0.0%	0.0%

**Figure 2:**
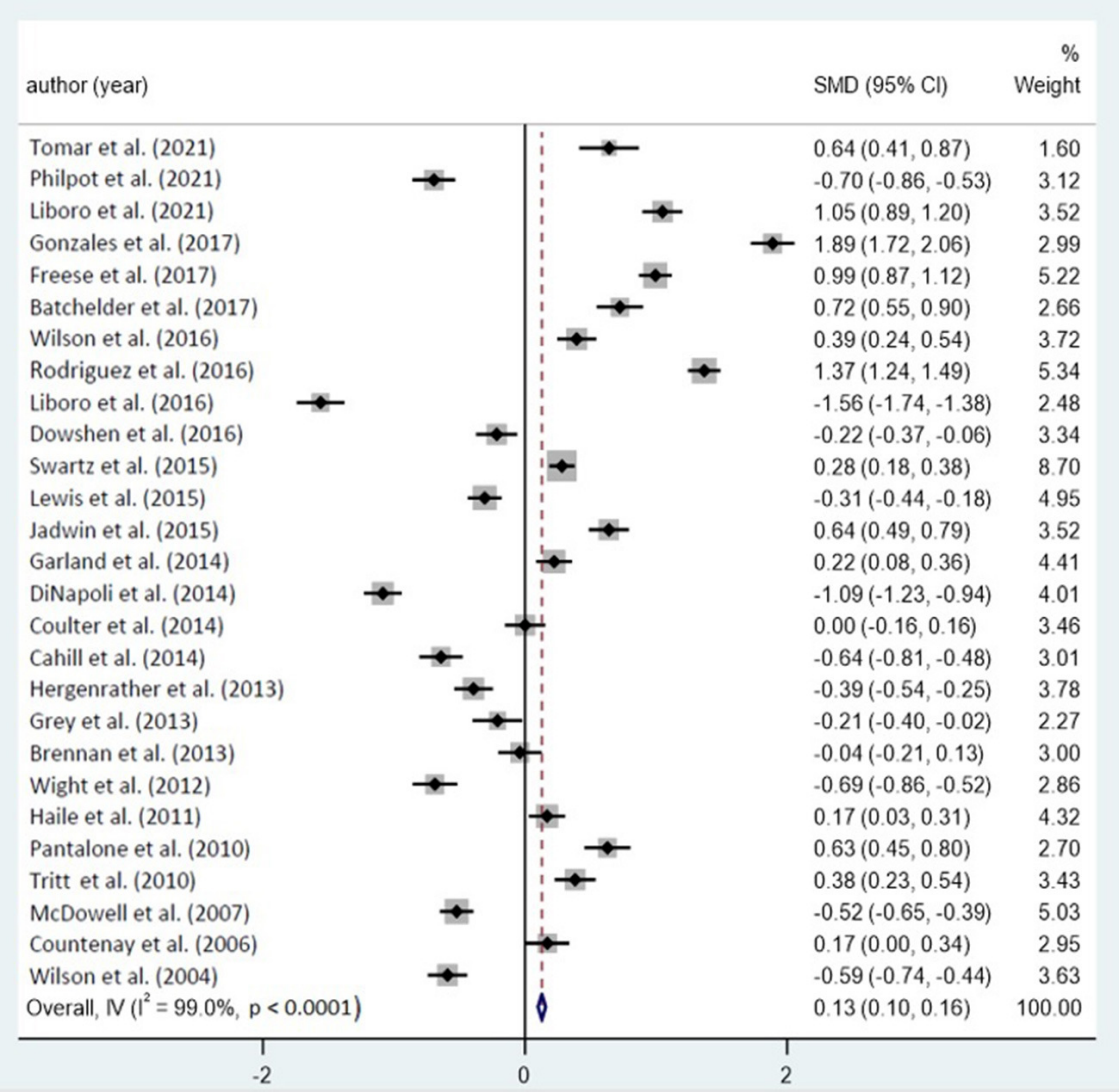
Forest plot with included studies on the effect of HIV/AIDS and mental health.

Cancer remains an untreatable illness in the present state of medical science. There is minimal research on the effect of inflammation and fibrosis on patients' mental wellbeing at the moment, which limits the volume of publications used for the meta-analysis. Prisma 2020 was used to conduct a search of the literature, and a few of those had issues including a huge sample size or a lack of clarity in the reporting of findings. Ultimately, 33 papers (Figure 2) were chosen for meta-analysis. The following are the findings:

Table 4 summarizes the findings of a meta-analysis on the potential effect of inflammation and fibrosis on the psychological wellbeing of LGBT sufferers. The SMD column in the table indicates the effect value that corresponds to the literature in the meta-analysis. All of the studies included in the meta-analysis are comparative studies where the control group is either ordinary or LGBT patients with cancer who have inflammation and fibrosis. Through looking at the mean scores on psychological wellbeing factors for the two groups, we can determine if cancer-related inflammation and fibrosis have a substantial effect on the psychological wellbeing of LGBT sufferers. The greater the value, the more dysfunctional the psychological state.

**Table 4:** Meta-analysis of mental health of cancer LGBT patients.

**Author(s) (year)**	**SMD**	**[95% Conf. interval]**	**[95% Conf. interval]**	**% Weight**	**Study quality**
Rhoten et al. (2022) (94)	12.48	10.05	14.92	0.97	Good
Feit et al. (2022) (95)	20.63	18.5	22.76	1.27	Good
Cheng et al. (2022) (96)	1.60	0.33	2.87	3.57	Good
Waters et al. (2021) (97)	−0.75	−2.23	0.73	2.61	Good
Sutter et al. (2021) (6)	20.25	18.57	21.94	2.02	Good
Skorzewska et al. (2021) (98)	−2.66	−3.96	−1.36	3.4	Moderate
Mulholand et al. (2021) (99)	−1.74	−2.83	−0.66	4.86	Good
Messona et al. (2021) (100)	11.68	10.34	13.02	3.19	Good
Drysdale et al. (2021) (101)	1.63	0.16	3.10	2.65	Good
Desai et al. (2021) (102)	7.88	6.64	9.12	3.76	Good
Cloyes et al. (2021) (103)	1.56	0.08	3.05	2.61	Moderate
Chidiac et al. (2021) (104)	−1.10	−2.60	0.40	2.55	Moderate
Burki et al. (2021) (105)	3.11	2.13	4.09	5.99	Good
Berner et al. (2021) (106)	7.93	6.28	9.58	2.12	Good
Austria et al. (2021) (107)	0.73	−0.55	2.01	3.52	Good
Sutter et al. (2020) (108)	2.08	0.92	3.24	4.25	Good
Sheeham et al. (2020) (109)	17.32	15.50	19.14	1.73	Good
Peitzmeier et al. (2020) (110)	9.36	7.55	11.16	1.76	Good
Ozkara et al. (2020) (111)	−1.63	−3.30	0.03	2.07	Moderate
Mclnnis et al. (2020) (112)	−8.77	−10.18	−7.36	2.9	Good
Kano et al. (2020) (113)	−13.42	−15.53	−11.30	1.29	Good
Haviland et al. (2020) (114)	1.54	0.49	2.59	5.21	Good
Grasso et al. (2020) (115)	0.91	−0.33	2.16	3.71	Good
Cattelan et al. (2020) (116)	8.57	7.29	9.84	3.54	Good
Berner et al. (2020) (117)	5.66	4.25	7.07	2.87	Good
Arnold et al. (2020) (118)	10.98	9.43	12.53	2.4	Good
Stevens et al. (2019) (119)	−7.97	−9.66	−6.29	2.02	Good
Schabath et al. (2019) (120)	20.94	19.03	22.84	1.58	Moderate
Rice et al. (2019) (121)	4.55	3.16	5.94	2.98	Good
Kamen et al. (2019) (122)	2.11	−1.67	2.48	2.99	Good
Cathcart et al. (2019) (123)	−4.59	−6.22	−2.97	2.17	Moderate
Tamargo et al. (2017) (124)	3.20	1.80	4.61	2.91	Good
Russell et al. (2016) (125)	8.14	7.02	9.26	4.57	Good
overall	12.48	10.05	14.92	100	

Test of overall effect = 0: z = 268.40, p < 0.0001.

SMD is the disparity in scores between the two categories, and the majority of research shows that cancer inflammation and fibrosis have a detrimental effect on the psychological wellbeing of LGBT cancer sufferers. According to the 33 research on the psychological wellbeing of cancer LGBT individuals examined in this study, the disparity in psychological wellbeing scores between cancer LGBT individuals and the general population varies between −8.77 and 20.94. The overall findings indicated that there was a 12.48 point gap in psychological wellbeing scores between the cancer LGBT subgroup and the general group (95% confidence interval was 10.05–14.92, egger Test *Z*-value = 268.40, *p* < 0.1).

While the techniques or ratings used to assess psychological wellbeing in such research vary throughout the publications, as a meta-analysis, this study excluded various intervention treatments. As shown in the preceding table, the ultimate overall effect *Z*-value = 268.40 and the *p* < 0.0001, suggesting that the mean psychological wellbeing score in the control group is 12.48 points higher compared to the experimental sample, and that is noteworthy. This demonstrates that cancer's inflammation and fibrosis have a substantial detrimental effect on the psychological wellbeing of the LGBT community.

**Figure 3:**
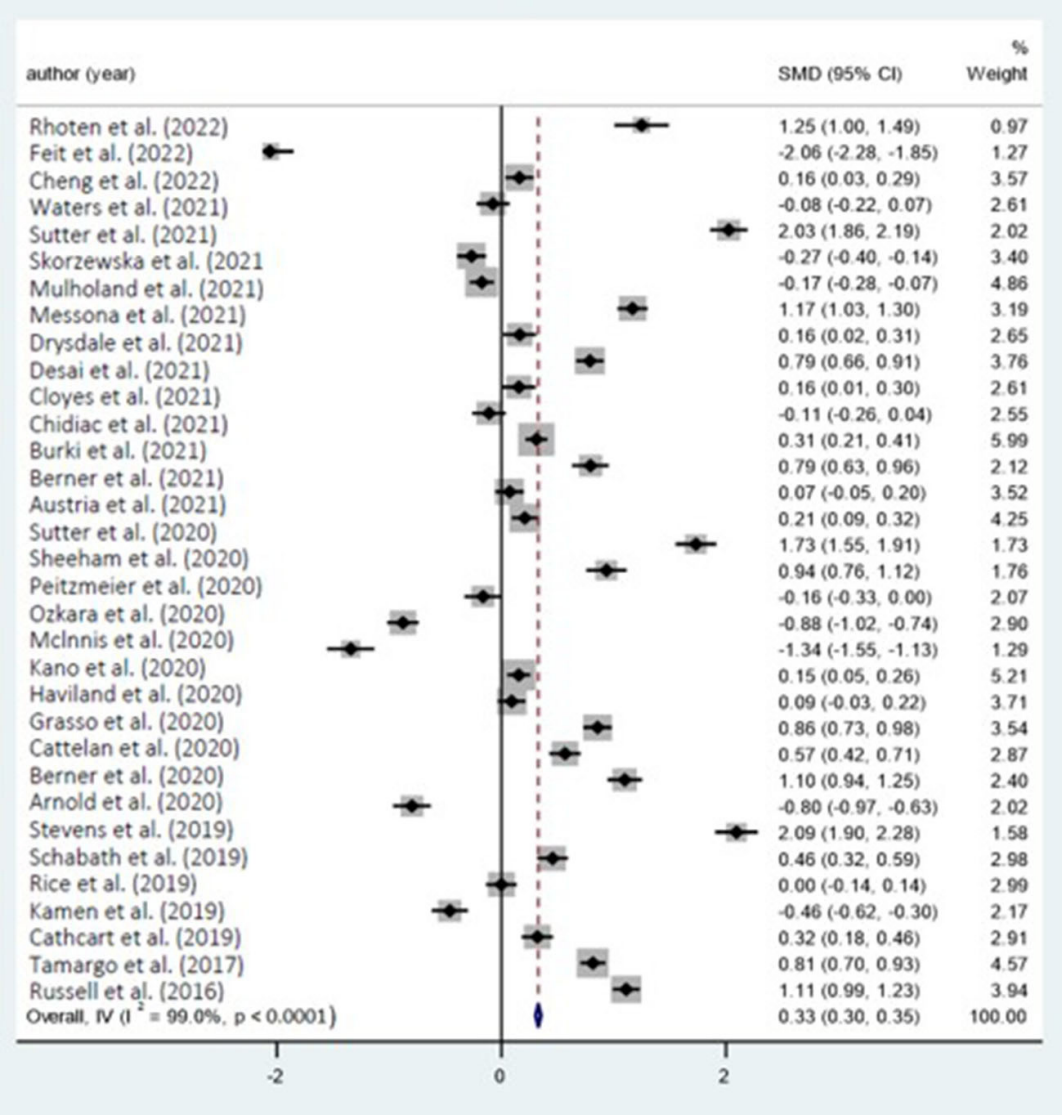
Forest plot with included studies on the cancer and mental health.

The preceding Table 5 summarizes the heterogeneity test outcomes from 33 analyses. Like the table indicates, the *p* < 0.0001, which is < 0.001, suggesting heterogeneity. The heterogeneity score is 99.0%, suggesting that available research has a high degree of heterogeneity. Eventually, the associated Cochran's *Q*-value is 3,300.34 and the accompanying *p* < 0.1, suggesting that inflammation and fibrosis in cancer will have a substantial detrimental effect on sufferers' psychological wellbeing (Figure 3).

**Table 5:** Heterogeneity analysis of related studies.

**Measure**	**Value**	***df***	***p*** **-value**
Cochran's Q	3,300.34	33	0.0001
H	10.001	1.000	
*I^2^* (%)	99.0%	0.0%	0.0%

## Discussion

Cancer and HIV/AIDS are significant illnesses affecting contemporary population wellbeing (20, 126). More effective technical strategies for HIV/AIDS and cancer do not exist at present. Cancer is often treated with surgery, radiation, and chemotherapy (127). The majority of cancer therapies, particularly chemotherapy, are very painful for sufferers. Unpleasant sensations have a direct effect on the psychological wellbeing of cancer sufferers, lowering their standard of living even more (128). For HIV/AIDS sufferers, the many difficulties created by social groups' intrinsic prejudice and immunity will have a major detrimental effect on their psychological wellbeing. Current research has examined the detrimental impact of inflammation and fibrosis on the psychological wellbeing of cancer and HIV/AIDS sufferers (129). Additionally, because of societal discrimination and marginalization, it is always challenging for LGBT communities to develop regular human ties, and as a result, they frequently struggle with autism and even depression, raising concerns about their psychological wellbeing (130). According to available studies, the effect of LGBT group psychological wellbeing issues, cancer, and HIV/AIDS on LGBT community psychological wellbeing has piqued the interest of numerous academics (131, 132). Nevertheless, actual studies on the psychological wellbeing of LGBT cancer and HIV/AIDS sufferers are scarce. The researcher examined just over a hundred datasets, including Google Academic and Web of Science. Twenty-seven meta-analyses evaluating the effect of inflammation and fibrosis on the psychological wellbeing of the LGBT community were performed, omitting certain prospective studies and inadequate data reporting. A meta-analysis of the effect of inflammation and fibrosis on LGBT community psychological wellbeing was conducted on 33 of them.

Based on the findings, the ultimate overall cancer test has a *Z-*value = 86.58 and a *p* < 0.0001. LGBT cancer sufferers had a substantially higher score than overall LGBT participants. Cancer-related inflammation and fibrosis have a detrimental effect on the psychological wellbeing of LGBT patients. Moreover, the ultimate HIV/AIDS overall test results show a *Z*-value of 268.40 and a *P* < 0.1, HIV/AIDS sufferers with LGBT scores are substantially higher than participants from the general LGBT category, and inflammation and fibrosis in cancer have a substantial adverse effect on the psychological wellbeing of LGBT sufferers.

## Results

Using a total of 48 entries from Google academic, the Web of Science, and the paper's citations, we discovered 370 articles that were connected to the research. Thirty-nine of the studies were retrospective, while the remaining 27 were prospective. They focused on 9,898 pieces of information regarding the psychological health of LGBT cancer patients and 14,465 cancer patients with HIV/AIDS.

We discovered through meta-analysis that the disparity in psychological wellbeing scores between HIV/AIDS LGBT individuals and the general population ranged between −10.86 and 15. The overall findings revealed a 1.270-point difference in psychological wellbeing scores between the HIV/AIDS LGBT group and the general population (95% confidence interval 0.990–1.560, *Z* = 86.58 and *P* < 0.1). The disparity in psychological wellbeing scores between cancer LGBT individuals and the general population ranges between −8.77 and 20.94, according to the 33 papers on the psychological wellbeing of cancer LGBT individuals examined for this study. The overall findings revealed a 12.48-point difference in psychological wellbeing scores between the cancer LGBT subgroup and the general population (95% confidence interval: 10.05–14.92; egger Test *Z*-value = 268.40; *P* < 0.1). The aforementioned findings indicate that the inflammation and fibrosis associated with HIV/AIDS and cancer have a significant negative impact on the psychological wellbeing of the LGBT community.

## Limitations

This research performed a meta-analysis of the effects of cancer and HIV/AIDS on LGBT sufferers' psychological wellbeing. To begin, it gathered pertinent research evidence through Google Academic and Web of Science. Due to the limited number of participants in this research and the tiny proportion of relevant publications, the researcher chooses 27 of them as meta-analysis samples for HIV/AIDS impact and 34 as meta-analysis samples for cancer impact. We could tell from meta-analysis that inflammation and fibrosis associated with cancer and HIV/AIDS have a substantial detrimental effect on the psychological wellbeing of LGBT people. The primary drawback of this paper is the minimal amount of literature used for the meta-analysis, and it is mostly attributable to the researcher's limited research subjects.

## Future implications

The needs and concerns reported by LGBT individuals in the studies included in this systematic review indicate the need for additional research on LGBT HIV/AIDS and cancer care policy and practice. It has been determined that LGBT individuals accept questions about their sexual orientation or gender identity in healthcare settings, which has a positive effect on their behaviors and attitudes regarding healthcare. Inclusive and reflective practitioners in the healthcare system who were proactive in their profession by, for example, recognizing the identities of LGBT people and providing them with specialized cancer care (133). The LGBT patients reported feeling safe and content with the cancer care they received. This suggests that LGBT cancer patients require interventions that are culturally competent. There is a need for care providers to be aware of the potential susceptibility of LGBT cancer patients to specific issues (53).

Concerning their mental health or identity disclosure, it is necessary to develop programs to educate care providers on their responsibility to assist SGM in their gender disclosure and assist them in overcoming adverse experiences. It has been suggested that inclusive language can foster a sense of safety and comfort in individuals who identify as SGM. However, if they wish to maintain their anonymity, this should also be respected (20). Regarding the LGBT community's healthcare disparities, we must direct programs to be cognizant of the issues and concerns they face. More effort is required to educate nurses and other health care professionals about patient care and meet the specific needs of LGBT HIV/AIDS and cancer patients.

Moreover, leadership styles such as transformational leadership and authentic leadership, as well as intersectionality, could be integrated into the cultural opportunities to structure clinical cancer care and address disparities in cancer care experienced by SGM populations. To further improve the health impartiality of SGM, clinicians and researchers require guidance and training regarding the culturally appropriate compilation of sexual orientation and gender identity data and the applicability of this information for cancer prevention, diagnosis, treatment, and survivorship in SGM.

## Conclusion

A meta-analysis was conducted to determine the impact of inflammation and fibrosis in HIV/AIDS and cancer on LGBT patients' psychological wellbeing. The study included 55 papers that were deemed relevant by the researcher, who then culled 27 for use in an HIV/AIDS meta-analysis and saved 33 for use in a cancer meta-analysis. The researcher then used stata.16 to evaluate the data. This analysis led to the following conclusions: The inflammation and fibrosis associated with HIV/AIDS and cancer have a negative impact on the psychological wellbeing of LGBT individuals. Moreover, the test for heterogeneity reveals that the findings of 30 publications exhibit substantial heterogeneity. This demonstrates that some research suggests that the effects of HIV/AIDS, inflammation and fibrosis on cancer are negligible, and this could be explained by the use of diverse methods for assessing psychological wellbeing. On the other hand, inflammation and fibrosis continue to have a major detrimental impact on the psychological wellbeing of LGBT individuals with cancer and HIV/AIDS.

## Data availability statement

The original contributions presented in the study are included in the article/supplementary material, further inquiries can be directed to the corresponding authors.

## Author contributions

Conceptualization: AC, LL, EY, PT and JL. Methodology: AC, LL, and JL. Validation: AC, JL, and JH. Formal analysis: AC, JL, and PT. Investigation and writing—original draft preparation: AC and JL. Data curation: HT, AC, JL, and JH. Visualization: AC, JL, JH, WH, AI, and PT. Supervision: PT and EY. All authors contributed to the article and approved the submitted version.

## Funding

The preparation of this manuscript was partially supported by the funding from the Department of Applied Social Sciences, The Hong Kong Polytechnic University, Hong Kong SAR, China, Research Grants Council of Hong Kong (General Research Fund 14106518, 14111019, 14111720, and Postdoctoral Fellowship Scheme PDFS2122- 4S06), State Key Laboratory of Translational Oncology, The Chinese University of Hong Kong's Faculty Innovation Award (4620528), Direct Grant for Research (4054510 and 4054668), and Postdoctoral Fellowship Scheme 2021-22 (NL/LT/PDFS2022/0360/22lt).

## Conflict of interest

The authors declare that the research was conducted in the absence of any commercial or financial relationships that could be construed as a potential conflict of interest.

## Publisher's note

All claims expressed in this article are solely those of the authors and do not necessarily represent those of their affiliated organizations, or those of the publisher, the editors and the reviewers. Any product that may be evaluated in this article, or claim that may be made by its manufacturer, is not guaranteed or endorsed by the publisher.
